# Recent Advances in the Domino Annulation Reaction of Quinone Imines

**DOI:** 10.3390/molecules29112481

**Published:** 2024-05-24

**Authors:** Zhen-Hua Wang, Xiao-Hui Fu, Qun Li, Yong You, Lei Yang, Jian-Qiang Zhao, Yan-Ping Zhang, Wei-Cheng Yuan

**Affiliations:** 1 Innovation Research Center of Chiral Drugs, Institute for Advanced Study, Chengdu University, Chengdu 610106, China; fuxh1367@126.com (X.-H.F.); youyong@cdu.edu.cn (Y.Y.); yanglei@cdu.edu.cn (L.Y.); zhaojianqiang@cdu.edu.cn (J.-Q.Z.); zhangyanping@cdu.edu.cn (Y.-P.Z.); 2 School of Materials and Environmental Engineering, Chengdu Technological University, Chengdu 611730, China; lqun1@cdtu.edu.cn; 3 College of Materials and Chemistry & Chemical Engineering, Chengdu University of Technology, Chengdu 610059, China

**Keywords:** quinone imines, domino reaction, annulation reaction, cyclic compound, organic synthesis

## Abstract

Quinone imines are important derivatives of quinones with a wide range of applications in organic synthesis and the pharmaceutical industry. The attack of nucleophilic reagents on quinone imines tends to lead to aromatization of the quinone skeleton, resulting in both the high reactivity and the unique reactivity of quinone imines. The extreme value of quinone imines in the construction of nitrogen- or oxygen-containing heterocycles has attracted widespread attention, and remarkable advances have been reported recently. This review provides an overview of the application of quinone imines in the synthesis of cyclic compounds via the domino annulation reaction.

## 1. Introduction

With the rapid development of medicinal and natural product chemistry, the diversity and complexity of organic molecules are increasing [1,2,3,4,5,6]. Therefore, developing efficient organic synthesis strategies to cope with this situation is very necessary. By exploring a series of reliable synthetic methods, it is possible to efficiently construct the structurally complex molecular frameworks found in natural products and biologically active compounds, including carbocyclic and heterocyclic structures, thereby facilitating the discovery of potential new drugs and pesticides [7,8,9,10,11,12]. Among the numerous reported synthetic strategies, domino reactions have attracted considerable attention due to their potential to conserve resources, reduce waste generation during the synthesis process, and align with the principles of green chemistry [13,14,15,16]. Most importantly, they enable the rapid assembly of polycyclic structures from simple starting materials [17,18,19,20,21,22]. This strategy has been successfully applied in the synthesis of natural products and bioactive compounds, demonstrating its potential to streamline the construction of complex molecular architectures [23,24,25,26]. By designing new synthons and optimizing domino reaction pathways, researchers can efficiently construct complex organic molecular structures and make breakthroughs in synthesizing natural products and drugs. In addition, domino reactions can provide ample space for developing new catalysts and reaction conditions to drive innovation and progress in organic synthesis. As domino reaction technology continues to be refined, new opportunities are emerging in organic synthesis.

**Figure 1:**
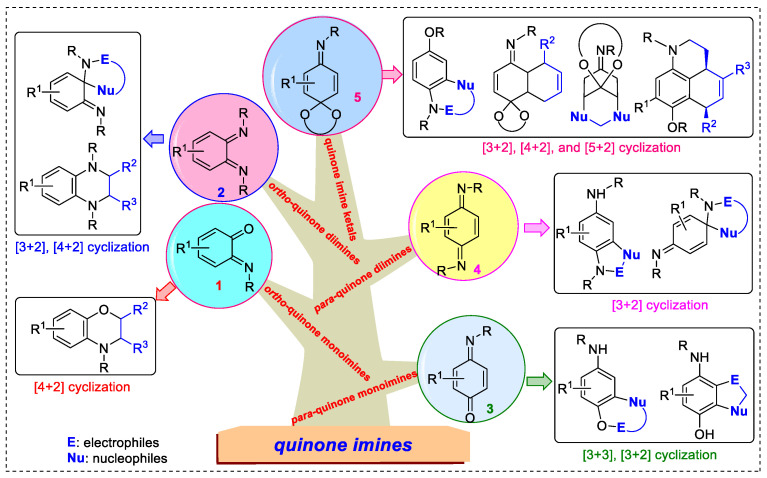
Overview of the quinone imines.

Quinones and their derivatives have attracted increasing attention in organic synthesis because of their wide applications in medicine, pesticides, dyes, energy storage, and various fine chemical products [27,28,29,30,31]. Quinone imines, as highly reactive electrophiles containing multiple active sites, can be used in aromatic functionalization, amination, and cyclization reactions, providing efficient tools and methods for synthetic chemistry [32,33,34,35,36,37,38,39]. In particular, annulation reactions involving quinone imines have been widely used to efficiently construct heterocycles, especially nitrogen- and oxygen-containing fused aromatic rings, providing an efficient method for the synthesis of complex molecules. The disclosed quinone imines mainly include *ortho*-quinone monoimines, *ortho*-quinone diimines, *para*-quinone monoimines, *para*-quinone diimines, and quinone imine ketals, which are defined by the number and locations of the imine groups attached to the quinone structure (Figure 1). The *ortho*-quinone monoimines can be used as aza-dienes for [4 + 2] annulation with alkenes or ketene enolates. The *ortho*-quinone diimines can be selected as imines to participate in [2 + n] cyclization reactions, and as 1,4-diazadienes to undergo [4 + n] cycloaddition reactions. The *para*-quinone monoimines commonly undergo [3 + 3] and [3 + 2] annulations. The *para*-quinone diimines are always used as C–C–N units to construct indole derivatives. The [3 + 2], [4 + 2], and [5 + 2] annulations can be achieved using quinone imine ketals as electrophilic species. Although the annulation reaction involving quinone imines has become an efficient platform for obtaining heterocyclic compounds, there is no comprehensive summary of this research area [40]. Therefore, a timely and relevant review on this topic is urgently needed, which is important for the future development of this field. Herein, for the first time, we discuss in detail the recent advances in the construction of cyclic compounds by the annulation reactions of quinone imines. This review is organized into five sections according to the types of quinone imines, including *ortho*-quinone monoimines, *ortho*-quinone diimines, *para*-quinone monoimines, *para*-quinone diimines, and quinone imine ketals.

## 2. Domino Reactions of *Ortho*-Quinone Imines

### 2.1. Domino Reaction of Ortho-Quinone Monoimines

**Scheme 1:**
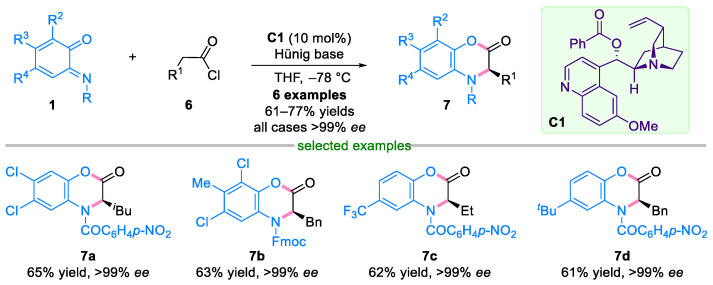
Asymmetric (4 + 2) cycloaddition of *ortho*-quinone monoimines and in situ generated ketene enolates.

In 2006, Lectka et al. developed an asymmetric [4 + 2] cycloaddition reaction involving *ortho*-quinone monoimines **1** and in situ generated ketene enolates (Scheme 1) [41]. In this report, the ketene enolate intermediates in situ generated from the reaction between benzoylquinidine **C1** and acid chlorides **6** underwent Michael addition to *ortho*-quinone monoimines **1**, leading to aromatization. Subsequently, intramolecular cyclization led to the formation of a series of 1,4-benzoxazines **7** in moderate yields and excellent enantioselectivities. Notably, the authors also disclosed a one-pot transformation to enable the highly stereoselective synthesis of chiral α-amino acid derivatives [42].

**Scheme 2:**
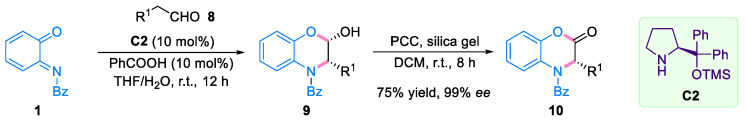
The asymmetric inverse electron-demand HDAR of aldehydes and *ortho*-quinone monoimines.

Soon after, Chen et al. also developed an organocatalytic enantioselective inverse-electron-demand hetero-Diels–Alder reaction (HDAR) of *ortho*-quinone monoimines **1** with aldehydes **8** (Scheme 2) [43]. The 1,4-benzoxazinones **10** were smoothly obtained with excellent stereoselectivities (up to 99% *ee*) after pyridinium chlorochromate (PCC) oxidation.

**Scheme 3:**
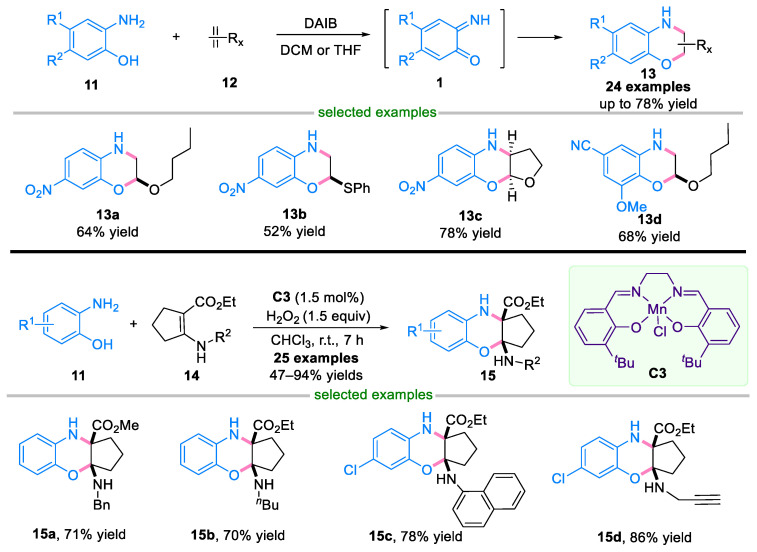
The oxidative [4 + 2] cycloaddition reaction between newly generated *ortho*-quinone monoimines and electron-rich olefins.

Another study on the construction of 1,4-benzoxazine derivatives based on *ortho*-quinone monoimines was reported by Peddinti et al. in 2012 (Scheme 3, top) [44]. In this research, highly reactive *ortho*-quinone monoimines **1** were in situ generated from *ortho*-aminophenol **11** by oxidation with diacetoxyiodobenzene (DAIB) as an oxidizing agent. The newly generated *ortho*-quinone monoimines **1** were then captured by vinylic (thio)ethers **12** to afford the desired 1,4-benzoxazine derivatives **13** in up to 78% yield. In 2020, Zhong et al., also developed an oxidative [4 + 2] cycloaddition of *ortho*-aminophenols **11** with cyclic enamines **14** (Scheme 3, bottom) [45]. For the mechanism, biomimetic Mn(III) catalyzed the oxidation of *ortho*-aminophenols **11** to furnish *ortho*-quinone monoimines **1**, which underwent the [4 + 2] cycloaddition with cyclic enamines **14** to give various tricyclic 1,4-benzoxazines **15** in up to 94% yield.

**Scheme 4:**
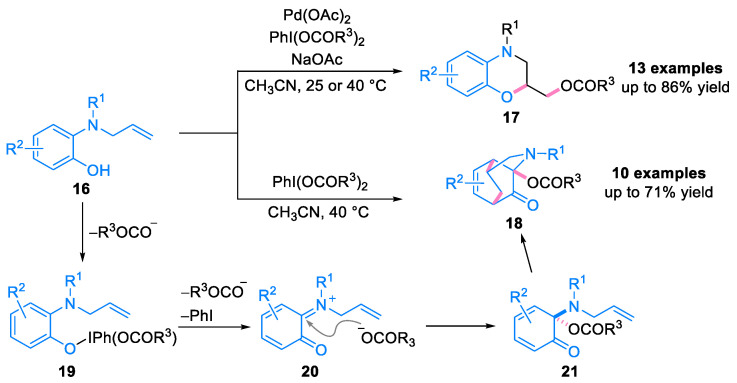
The switchable oxidative reactions of *N*-allyl-2-aminophenols.

**Scheme 5:**
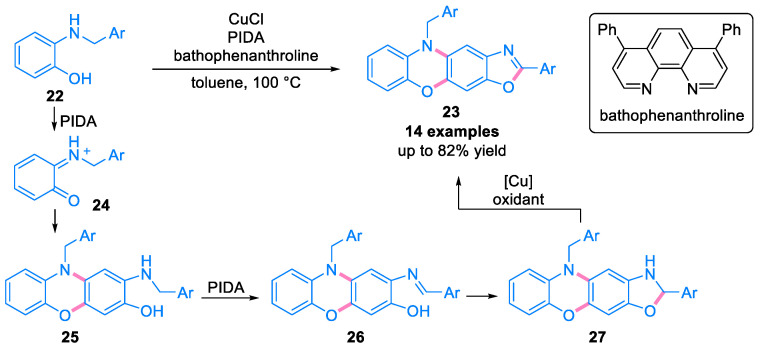
The oxidative dimerization/cyclization of 2-benzylaminophenols.

In 2021, Beccalli et al. disclosed a divergent oxidative cyclization of in situ generated *ortho*-quinone monoimines (Scheme 4) [46]. Selecting hypervalent iodines as the oxidant, Pd(OAc)_2_ enabled 6-exo-trig cyclization involving *N*-allyl-*N*-tosyl 2-aminophenol **16** to afford functionalized dihydro-1,4-benzoxazines **17** in a generally good yield. In the absence of a palladium catalyst, sequential nucleophilic addition and intramolecular Diels–Alder reactions gave a functionalized tricyclic system **18** in up to 71% yield. The present protocol featured that the oxidant acted as both a nucleophilic donor and an oxidizing agent. The following year, the group of Broggini developed a copper-catalyzed dimerization/cyclization reaction involving aminophenols (Scheme 5) [47]. The authors proposed that the *ortho*-quinone-type intermediates **24**, generated in situ from aminophenols **22** via phenyliodine diacetate (PIDA) oxidation, underwent a cyclization reaction with 2-benzylamino-phenols **22** to form the key intermediates **25**. According to the proposed mechanism, the intermediates **25** could also be produced through an alternative pathway (not shown). The intermediates **25** was further oxidized by PIDA to generate the intermediates **26**, followed by intramolecular cyclization and oxidation reactions of the intermediates **26**, ultimately furnishing the 5*H*-oxazolo[4,5-*b*]phenoxazine compounds **18** in up to 82% yield.

### 2.2. Domino Reaction of Ortho-Quinone Diimines

The *ortho*-quinone diimines are a class of structurally stable variants that possess structural motifs including diene, imine, and 1,4-diazadiene. Based on these features, they can serve as arylation reagents for 1,4-conjugate addition [48,49], as imines to participate in [2 + n] cyclization reactions, and as 1,4-diazadienes to undergo [4 + n] cycloaddition reactions.

**Scheme 6:**
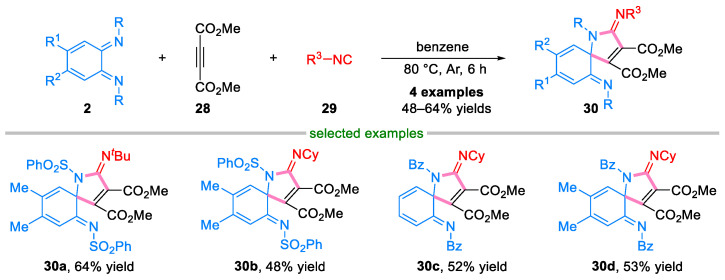
The three-component [3 + 2] cycloaddition involving *ortho*-quinone diimines. Cy, cyclohexyl.

There is only one study using *ortho*-quinone diimines as imines to participate in a domino reaction. In 2005, Nair et al. developed a three-component [3 + 2] cycloaddition reaction of *ortho*-quinone diimines, dimethyl acetylenedicarboxylate (DMAD), and isocyanates, which led to the construction of the spiroiminolactam derivatives **30** in moderate yields (up to 64%) (Scheme 6) [50]. In the transformation, the reaction between DMAD **28** and isocyanate **29** smoothly generated a zwitterionic intermediate, which then underwent a 1,3-dipolar cycloaddition reaction with the imine group of the *ortho*-quinone diimine to furnish spiroiminolactam.

**Scheme 7:**
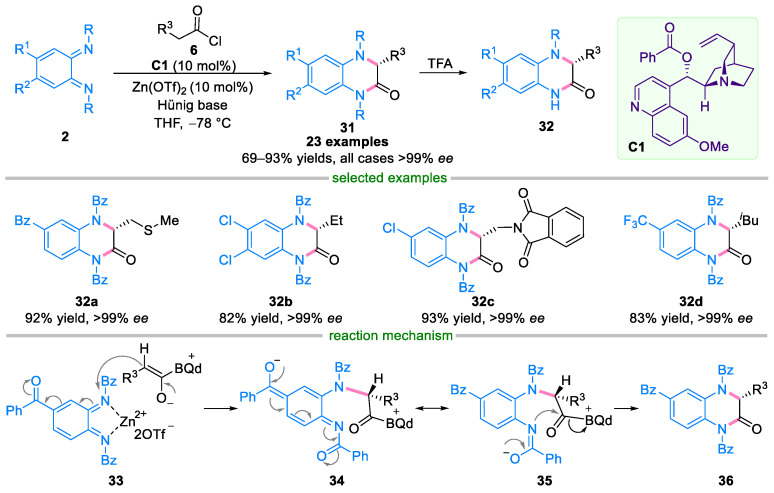
The asymmetric [4 + 2] cycloaddition reactions of *ortho*-quinone diimines and in situ generated ketene enolates.

The main application of *ortho*-quinone diimines **2** is mainly focused on using them as 1,4-diazadienes to participate in [4 + 2] cycloaddition for constructing dihydroquinoxaline derivatives. In 2006, Lectka et al. successfully developed the asymmetric [4 + 2] cycloaddition reaction between *ortho*-quinone diimines **2** and acid chlorides **6** (Scheme 7) [51]. In the reaction process, benzoylquinidine **C1** and Hünig’s base cooperatively activated the acid chlorides **6** to generate ketene enolates, which then underwent [4 + 2] cycloaddition with Lewis acid-activated *ortho*-quinone diimines **2**, resulting in the formation of biologically active quinoxalinone derivatives **31** with excellent stereoselectivities (all cases >99% *ee*). It is worth mentioning that the selective removal of nitrogen protecting groups could be achieved by trifluoroacetic acid (TFA) to furnish the compounds **32**.

**Scheme 8:**
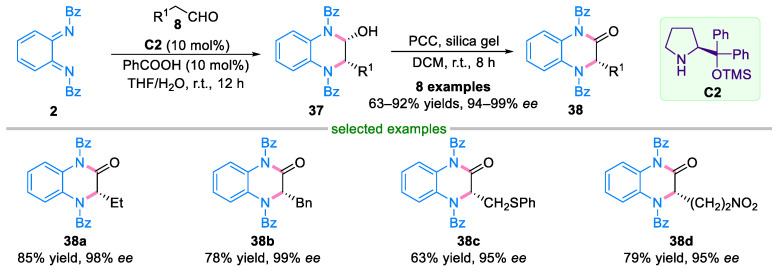
Organocatalytic enantioselective inverse-electron-demand HDAR of aldehydes and *ortho*-quinone diimides. Bz, benzoyl.

**Scheme 9:**
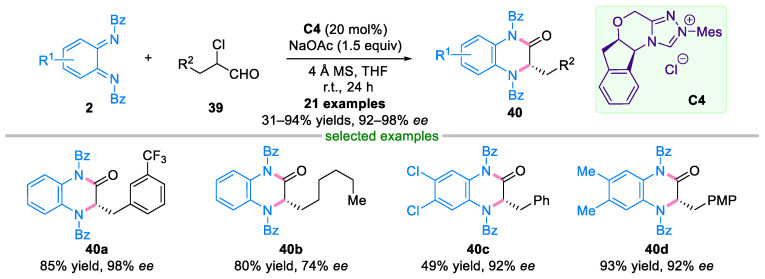
Chiral *N*-heterocyclic carbene-catalyzed [4 + 2] cycloaddition reaction between α-chloroaldehydes and *ortho*-quinone diimides.

In 2009, Chen et al. reported the asymmetric inverse-electron-demand HDAR between *N*-benzoyl *ortho*-quinone diimine **2** and aldehydes **8** (Scheme 8) [39]. Under the catalysis of proline-derived siloxane **C2** and benzoic acid, the reaction exhibited excellent enantioselectivities (95–99% *ee*). Another cyclization reaction of *ortho*-quinone diimines **2** with aldehydes **39** was disclosed in 2019 (Scheme 9) [52]. In this report, the chiral *N*-heterocyclic carbene **C4** activated α-haloaldehydes **39** to generate enol intermediates, which then underwent [4 + 2] cycloaddition reactions with *ortho*-quinone diimines **2** to give chiral dihydroquinoxaline products **40** with generally excellent *ee* values (92–98%).

**Scheme 10:**
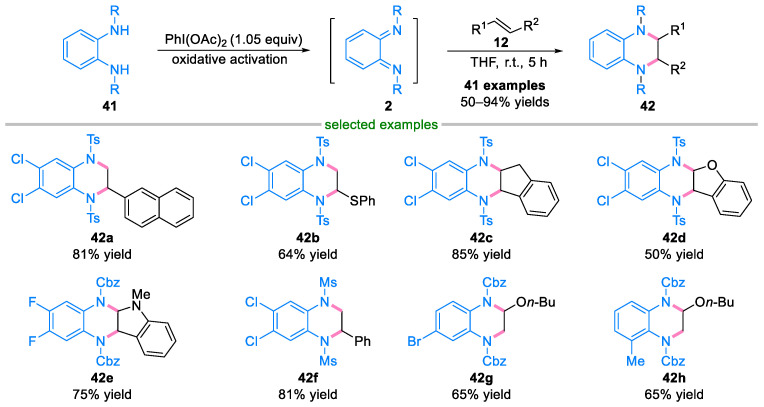
The [4 + 2] cyclization reaction between in situ formed *ortho*-quinone diimines and electron-rich olefins.

The cycloaddition reaction of in situ generated *ortho*-quinone diimines represents a powerful tool for streamlining the synthesis of functionalized tetrahydroquinoxalines and has potential applications in the construction of nitrogen-containing heterocycles. Recently, Zhong et al. employed an in situ oxidative activation strategy to accomplish a [4 + 2] cyclization reaction between *ortho*-phenylenediamine **41** and alkenes **12** (Scheme 10) [53]. This transformation is compatible with a range of alkene derivatives, such as styrenes, vinylic (thio)ethers, benzofurans, and indoles, affording a series of tetrahydroquinoxaline derivatives **42** in up to 94% isolated yield.

**Scheme 11:**
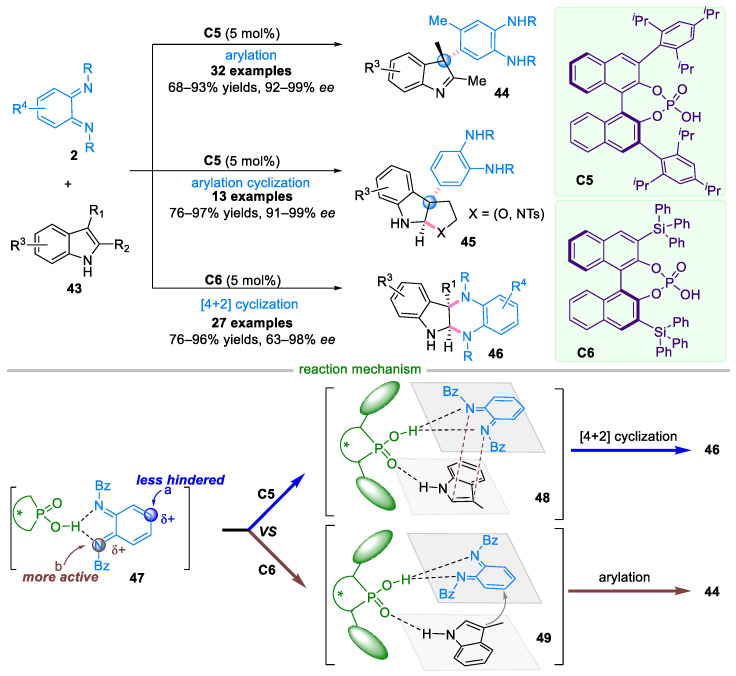
Diverse transformation of indole derivatives with *ortho*-quinone diimides. * represents that the skeleton is chiral.

More recently, Mei et al. reported, for the first time, the diversity-oriented catalytic asymmetric dearomatization of indoles **43** through reacting with *ortho*-quinone diimides **2** (Scheme 11) [54]. When 2,3-dimethylindoles were involved in the asymmetric dearomatization with chiral phosphoric acid **C5** as the catalyst, the arylation reaction afforded the products **44** with high reactivity and excellent stereoselectivity (68–93% yields, 92–99% *ee*). The reaction of tryptophols/tryptamines with *ortho*-quinone diimides could also be realized via a sequential dearomatization–cyclization process, leading to polycyclic indoline skeletons **45** that are widely present in biologically active compounds. Moreover, the dearomatic [4 + 2] cycloaddition reactions between *ortho*-quinone diimides and 3-substituted indoles were facilitated by chiral phosphoric acid **C6**. This transformation proceeded with high yields and excellent stereoselectivities, resulting in the fused indolines **46** in 76–96% yields with 63–98% *ee*.

## 3. Domino Reactions of *Para*-Quinone Imines

### 3.1. Domino Reaction of Para-Quinone Monoimines

**Scheme 12:**
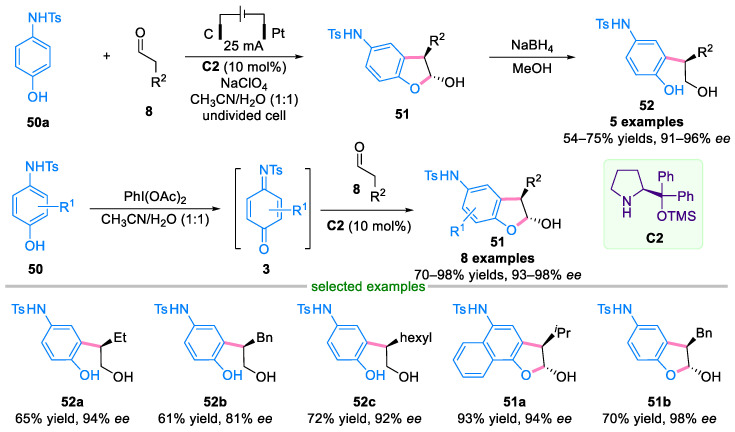
The enantioselective [3 + 2] cycloaddition of aldehydes with in situ formed *para*-quinone monoimines.

In 2010, Jørgensen et al. presented the [3 + 2] cycloaddition of aldehydes with in situ formed *para*-quinone monoimines by combining electrocatalysis and asymmetric organic catalysis (Scheme 12) [55]. Anodic oxidation of *N*-toluenesulfonyl-4-aminophenol **50a** proceeded smoothly to give *para*-quinone monoimine. The in situ generated *para*-quinone monoimine reacted with aldehydes **8** under the catalysis of proline-derived siloxane **C2** to furnish corresponding products **51**, which smoothly converted into the final products **52** by treatment with NaBH_4_. In addition, the developed transformation could also be achieved via the chemical oxidation process in 70–98% yields with 93–98% *ee* values.

**Scheme 13:**
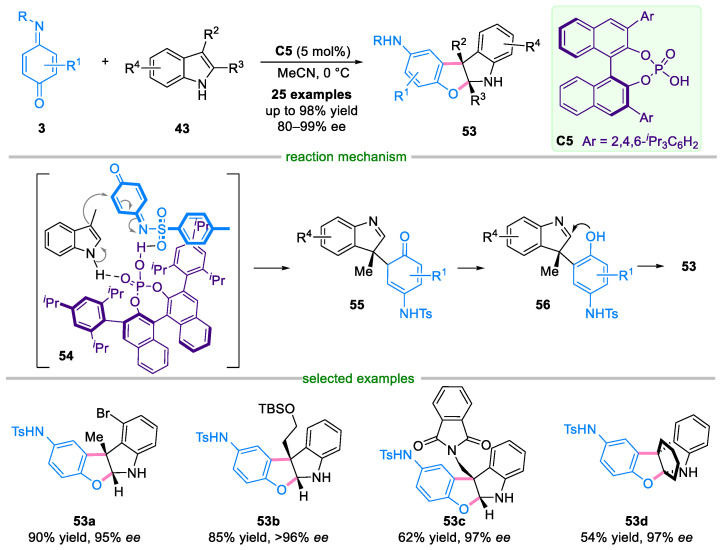
Chiral phosphoric acid catalyzed enantioselective [3 + 2] cycloaddition of substituted indoles with *para*-quinone monoimines.

In 2014, Zhang et al. pioneered the asymmetric domino cyclization reaction involving *para*-quinone monoimines (Scheme 13) [56]. Under the catalysis of chiral phosphoric acid **C5**, 3-substituted indoles **43** underwent an asymmetric [3 + 2] cyclization reaction with *para*-quinone monoimines **3**, successfully affording a series of benzofuroindoline derivatives **53** with high stereoselectivities (up to 99% *ee*). This transformation features that the bifunctional phosphoric acid **C5** activated both the 3-methylindoles and the *para*-quinone monoimines (shown as **54**). The 3-substituted indoles attacked the *para*-quinone monoimines from the *Re* face to give the intermediates **55**, which were promptly aromatized to produce the phenol intermediates **56**. Subsequently, an intramolecular cyclization occurred to generate the final products **53**.

**Scheme 14:**
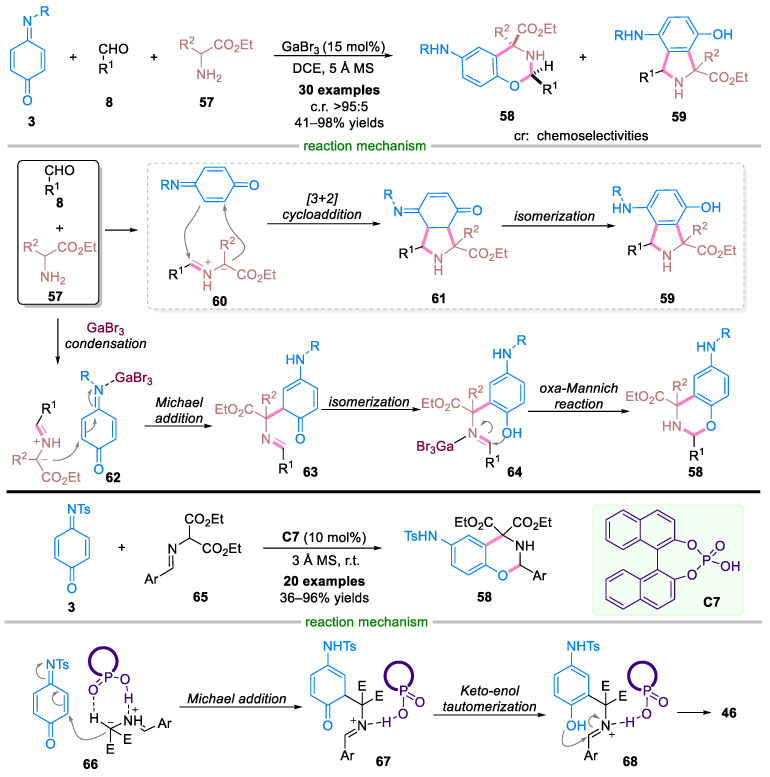
The [3 + 3] cycloadditions of in situ generated azomethine ylides with *para*-quinone monoimines.

In 2015, Shi et al. successfully developed a three-component [3 + 3] cycloaddition reaction involving *para*-quinone monoimines, aldehydes, and amino-esters (Scheme 14, top) [57]. Under the catalysis of GaBr_3_, the condensation of aldehydes **8** with amino-esters **57** resulted in the formation of azomethine ylides **60**, which completed the Michael addition reaction with *para*-quinone monoimines to form intermediates **63**. After keto-enol tautomerization, the generated intermediates **64** underwent intramolecular cyclization reactions, leading to the formation of dihydrobenzoxazine derivatives **58** in 41–98% yields. The in situ generated azomethine ylides **60** might also undergo a formal [3 + 2] cycloaddition process with the C=C bond of *para*-quinone monoamines to give compounds **59** but not the major products. Subsequently, Guo and his collaborators also reported the [3 + 3] cycloaddition reaction between *para*-quinone monoimines **3** and the azomethine ylide precursor **65** using racemic binaphthol-derived phosphoric acid **C7** as a catalyst, in which the transformation furnished dihydrobenzoxazine derivatives **58** in up to 96% yield (Scheme 14, bottom) [58].

**Scheme 15:**
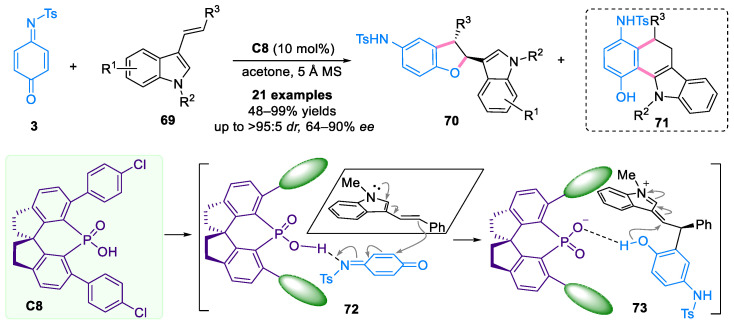
The chiral phosphoric acid-catalyzed [3 + 2] cycloadditions of *para*-quinone monoimine with 3-vinylindoles.

**Scheme 16:**
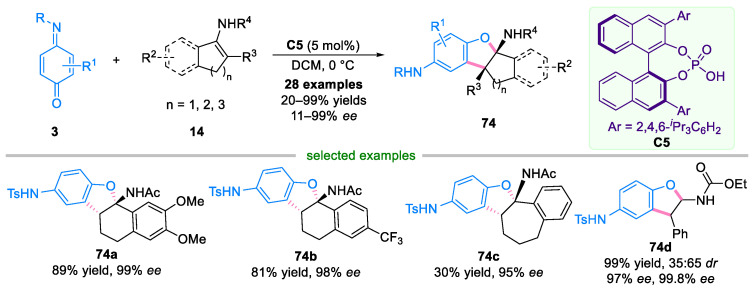
The asymmetric [3 + 2] cyclization reaction between *para*-quinone monoimines and cyclic enamines.

The Shi group also demonstrated the catalytic asymmetric [3 + 2] cycloaddition of *para*-quinone monoimine **3** with 3-vinylindoles **69** (Scheme 15) [59]. The cyclization products **70** were obtained in generally high yields with good to excellent stereoselectivities (up to 99% yield, 95:5 *dr*, 96:4 *er*), and no formal [4 + 2] cyclization products **71** were observed. In the reaction process, the spiro-chiral phosphoric acid **C8** promoted the enantioselective vinylogous Michael addition of 3-vinylindoles **69** to *para*-quinone monoimines **3** via the transition state **72** and formed the transient intermediate **73**, which then underwent intramolecular oxa-Michael addition to give the chiral indole-based 2,3-dihydrobenzofuran derivatives **70**. In the same year, Zhang et al. developed the asymmetric [3 + 2] cyclization reaction between *para*-quinone monoimines **3** and cyclic enamines **14** under the catalysis of chiral phosphoric acid **C5** (Scheme 16) [60]. Various polycyclic 2,3-dihydrobenzofurans **74** were obtained in moderated to excellent enantioselectivities (11–99% *ee*). In their report, the acyclic enamines could also undergo the desired transformation but exhibited very poor diastereoselectivities. Moreover, the *para*-quinone monoimine or cyclic enamine bearing a methyl group was not suitable for the developed protocol (not shown).

**Scheme 17:**
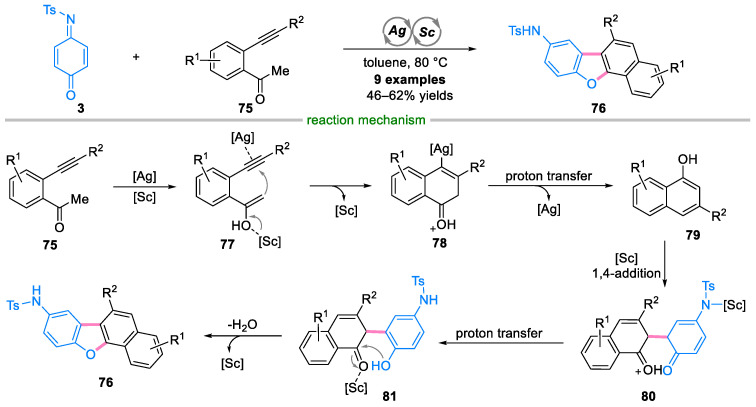
Silver/scandium-catalyzed transformation involving *para*-quinone monoimines and β-alkynyl ketones.

Co-catalysis involves the collaborative action of two or more catalysts to enhance a chemical reaction. These catalysts can carry out distinct functions, such as triggering different substrates, expediting various reaction steps, or boosting the effectiveness. By working in tandem reaction, co-catalysis often results in an increased reaction speed, selectivity, and overall efficacy compared to using a single catalyst [61]. Jiang et al. developed the first example of an Ag/Sc-catalyzed transformation involving *para*-quinone monoimine (Scheme 17) [62]. The disclosed reaction features an Ag/Sc-catalyzed 6-endo-dig cyclization reaction of aromatic *ortho*-alkynyl ketones **75** to furnish intermediates **78**, which underwent a proton transfer to give 1-naphthols **79** with simultaneous release of the Ag catalyst. The formed 1-naphthols **79** underwent a 1,4-addition reaction with *para*-quinone monoimines to give intermediates **80**, which then aromatized, followed by an intramolecular cyclization and dehydrogenation, finally providing tetracyclic naphtho[1,2-*b*]benzofurans **76** in moderate yields (46–62%).

**Scheme 18:**
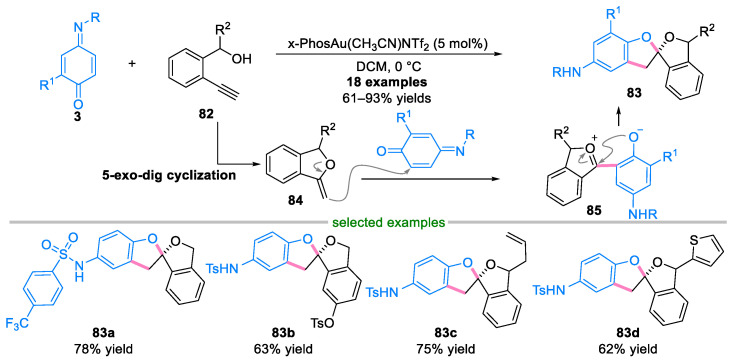
Gold-catalyzed cycloisomerization-spiroketalization of 2-ethynylbenzyl alcohol with *para*-quinone monoimines.

Spiroketal moieties are commonly found in natural products and pharmaceutical compounds, and they can impart unique biological characteristics and chemical reactivity to molecules. Xu et al. first developed the synthesis of spirocyclic compounds with a spiroketal skeleton by using *para*-quinone imines as three-atom building blocks (Scheme 18) [63]. Under the action of a gold catalyst, 2-ethynylbenzyl alcohol **82** underwent intramolecular 5-exo-dig cyclization to form enol ether intermediates **84**. The Michael addition of intermediates **84** to *para*-quinone monoimines **3** afforded intermediates **85**, followed by intramolecular cyclization to generate the desired 5,5-benzannulated spiroketals **83** in up to 93% yield.

**Scheme 19:**
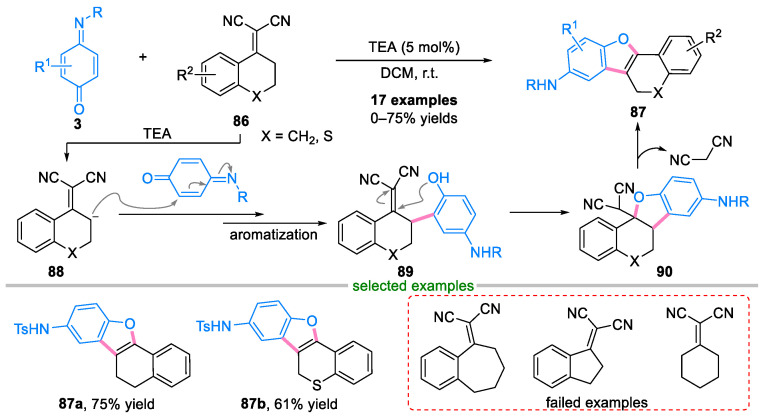
TEA-catalyzed [3 + 2] cyclization-elimination cascade of α,α-dicyanoolefins with *para*-quinone monoimines.

Pterocarpen derivatives exhibit a wide range of biological activities, including anti-HCV and antiestrogen properties [64,65,66]. Therefore, the efficient construction of these compounds has increasingly attracted the attention of synthetic chemists. In 2019, Zhang et al. presented the efficient synthesis of a novel class of pterocarpen analogs **87** through a [3 + 2] cyclization–elimination reaction between *para*-quinone monoimines **3** and α,α-dicyanoolefins **86** (Scheme 19) [67]. Using triethylamine (TEA) as a catalyst, the α,α-dicyanoolefins **86** underwent a Michael addition reaction with *para*-quinone imines **3**, followed by aromatization to generate intermediates **89**. Subsequently, the intramolecular cyclization reaction occurred to form the intermediates **90**, which then eliminated malononitrile to produce the final products **87** in up to 75% yield. It should be noted that a benzo-five-membered ring, benzo-seven-membered ring, and 2-cyclohexylidenemalononitrile did not react with the *para*-quinone imine under standard conditions.

**Scheme 20:**
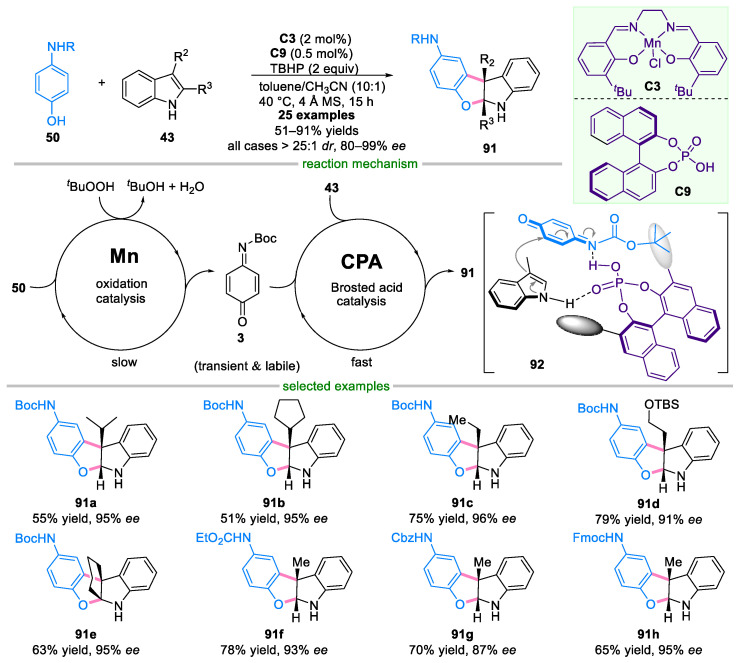
Asymmetric [3 + 2] cycloaddition reaction between in situ generated *para*-quinone monoimines and substituted indoles. Cbz, benzyloxycarbonyl. Fmoc, 9-fluorenylmethoxycarbonyl.

The first example of an asymmetric cycloaddition reaction between *para*-quinone monoimines generated by in situ oxidation and substituted indoles **43** was demonstrated by Zhong et al. (Scheme 20) [68]. In this report, the (salen)Mn(III) complex **C3** was used as a biomimetic surrogate of the metallocofactor to accomplish the in situ oxidation of 4-hydroxyanilines **50** for generating transient *para*-quinone monoimines **3**. Subsequent catalysis by chiral phosphoric acid **C9** induced the annulation of *para*-quinone monoamine **3** with substituted indoles **43**, resulting in the formation of chiral benzofuroindoline derivatives **91** in moderate to excellent yields with excellent stereoselectivities.

**Scheme 21:**
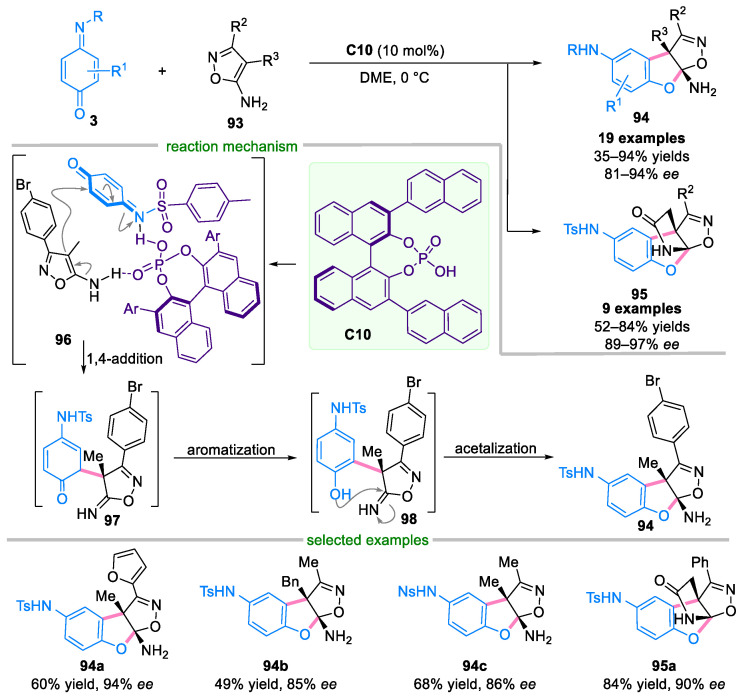
Chiral phosphoric acid-catalyzed enantioselective dearomative [3 + 2] annulation of 5-amino-isoxazoles with *para*-quinone monoimines.

**Scheme 22:**
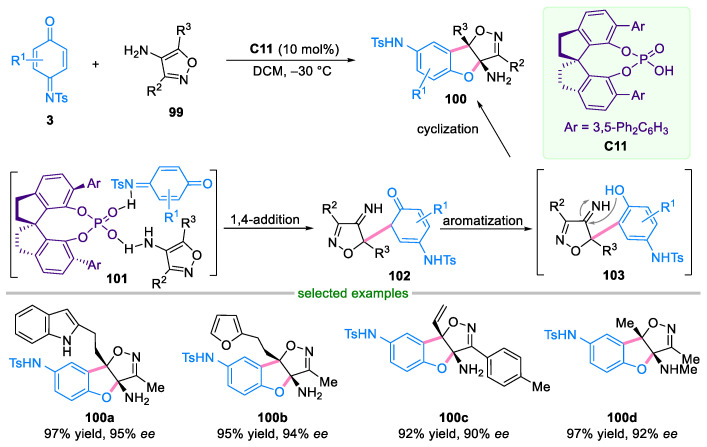
Chiral phosphoric acid-catalyzed enantioselective dearomative [3 + 2] annulation of 4-amino-isoxazoles with *para*-quinone monoimines.

The asymmetric dearomatization reaction, one of the efficient approaches for the synthesis of chiral heterocycles, has received wide attention from chemists [69,70,71]. Over the past 10 years, a variety of aromatic compounds, including naphthol, indole, benzofuran, and benzothiophene, have been used in asymmetric dearomative reactions. In contrast, the asymmetric dearomative cyclization of isoxazoles has only recently been achieved. In 2020, the Zhang group first reported the chiral phosphoric acid-catalyzed asymmetric dearomative cyclization reaction of 5-amino-isoxazoles **93** (Scheme 21) [72]. Chiral phosphoric acid **C10** catalyzed the enantioselective dearomative [3 + 2] annulations between 5-amino-isoxazoles **93** and *para*-quinone monoimines **3** in 1,2-dimethoxyethane (DME) at 0 °C to give the corresponding polycyclic compounds **94**. Furthermore, the reactions involving ethyl 4-acetate-isoxazol-5-amines and *para*-quinone monoimines afforded the bridged polycyclic scaffolds **95** in moderate yields with high *ee* values. Recently, the Zhang group also disclosed the dearomative cyclization reaction of 4-amino-isoxazoles **99** (Scheme 22) [73]. Similar to their previous report, highly enantioselective [3 + 2] annulation of 4-amino-isoxazoles **99** with *para*-quinone monoimines **3** was achieved under the catalysis of chiral phosphoric acid **C11**, providing access to structurally diverse isoxazoline-fused dihydrobenzofurans **100** with generally excellent enantioselectivities.

**Scheme 23:**
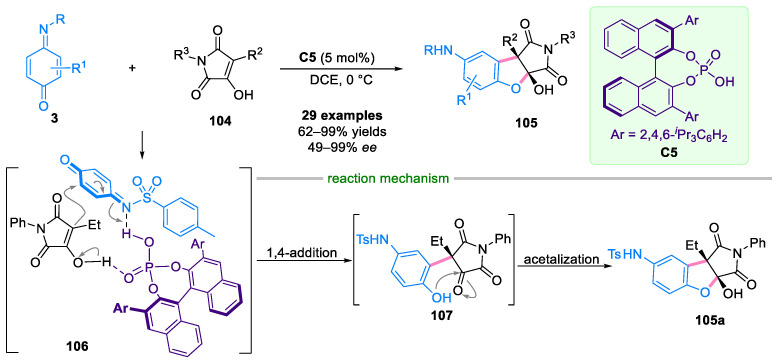
Chiral phosphoric acid-catalyzed enantioselective [3 + 2] annulation of 3-hydroxymaleimides with *para*-quinone monoimines.

In 2021, Zhang et al. also demonstrated an enantioselective [3 + 2] annulation involving *para*-quinone monoimines **3** and 3-hydroxymaleimides **104** (Scheme 23) [74]. The chiral phosphoric acid **C5** catalyzed the transformation to produce fused succinimide and dihydrobenzofuran **105** with generally excellent results (up to 99% yield, 99% *ee*).

**Scheme 24:**
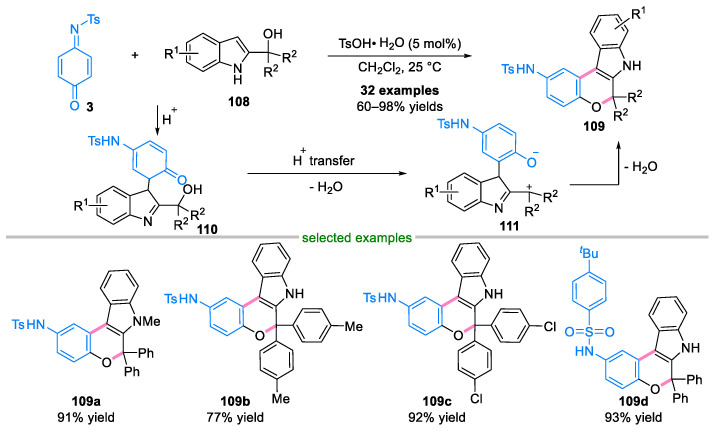
The formal [3 + 3] cyclization reaction of 2-indolylmethanols with *para*-quinone monoimines.

The [3 + 3] cyclization reaction involving *para*-quinone monoimines was reported by Zhen et al. in 2021 (Scheme 24) [75]. In their report, 2-indolylmethanols **108** acted as nucleophiles at the C3-position attacking *para*-quinone monoimines **3** to form the intermediates **110**, which underwent proton transfer and loss of water to form the intermediates **111**. A final intramolecular nucleophilic attack led to the formation of the desired cyclization products **109** in good to excellent yields.

**Scheme 25:**
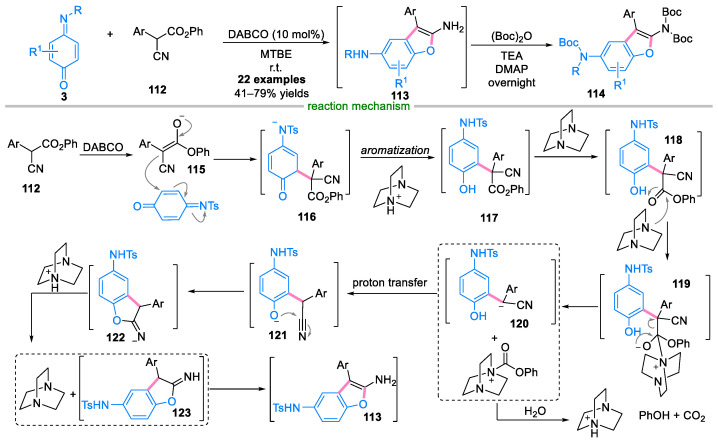
DABCO-catalyzed the formal [3 + 2] cyclization reaction of *para*-quinone monoimines with α-cyano arylacetates.

Zhang et al. also developed a divergent reaction between *para*-quinone monoimines **3** and α-cyano-α-arylacetates **112**. In 2019, they found that tandem conjugate addition and C–O ester migration occurred in refluxing acetonitrile to give various 2,2-diarylacetonitriles in generally good yields (not shown) [76]. In 2021, they found that an organic base could also promote the [3 + 2] cycloaddition reaction of *para*-quinone monoimines **3** with α-cyano-α-arylacetates **112** but resulted in the formation of 2-aminobenzofuran **113**, which was protected with di-*tert*-butyl dicarbonate to give **114** in moderate to good yields (Scheme 25) [77]. For the reaction mechanism, 1,4-diazabicyclo[2.2.2]octane (DABCO) promoted the deprotonation of phenyl α-cyano arylacetates **112** to give enolates **115**. Nucleophilic addition of the enolates **115** to *para*-quinone monoimines followed by aromatic rearrangement and protonation led to the formation of intermediates **117**. Then, the nucleophilic addition of DABCO to intermediates **117** gave zwitterions **119**, and subsequent C−C cleavage and intramolecular proton transfer generated intermediates **121**. Finally, the intramolecular nucleophilic addition of the phenoxy anion to the nitrile group led to the cyclization process to form intermediates **123**, which then isomerized to form the final products **113**.

**Scheme 26:**
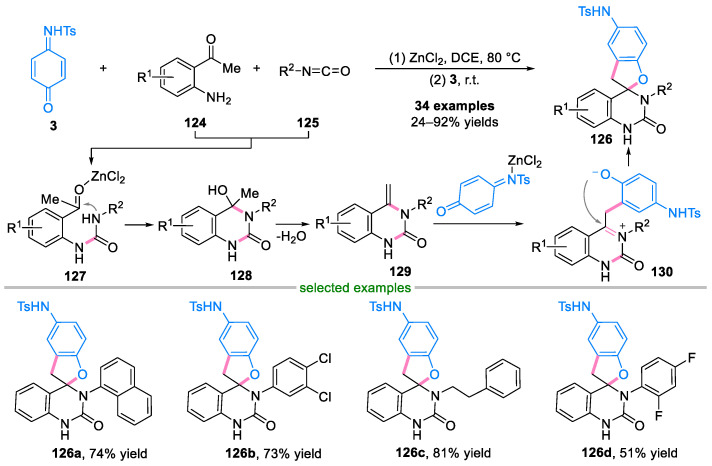
ZnCl_2_-catalyzed three-component cyclization reaction of *para*-quinone monoimines, 2-aminoacetophenones, and isocyanates.

In 2022, Xu et al. reported the successful construction of spiro-fused 2,3-dihydrobenzofurans **126** via a one-pot three-component cyclization reaction of *para*-quinone monoimines, 2-aminoacetophenones, and isocyanates (Scheme 26) [78]. Under Lewis acid catalysis, the [4 + 2] cyclization reaction of 2-aminoacetophenones **124** and isocyanates **125** smoothly generated an intermediate **129**, which subsequently underwent a domino [3 + 2] cyclization reaction with *para*-quinone monoimines **3**, affording the spiro-fused 2,3-dihydrobenzofurans **126** in a maximum yield of 92%.

### 3.2. Domino Reaction of Para-Quinone Diimines

Cyclization reactions involving *para*-quinone diimines are commonly used to construct nitrogen-containing heterocycles. Currently, the cyclization reactions of *para*-quinone diimines are mainly categorized into two types: one involving their use as the C–N unit in [3 + 2] cycloaddition reactions to construct spirocyclic compounds, and the other involving their participation as the C–C–N unit in [3 + 2] cycloaddition reactions to construct polycyclic compounds.

**Scheme 27:**
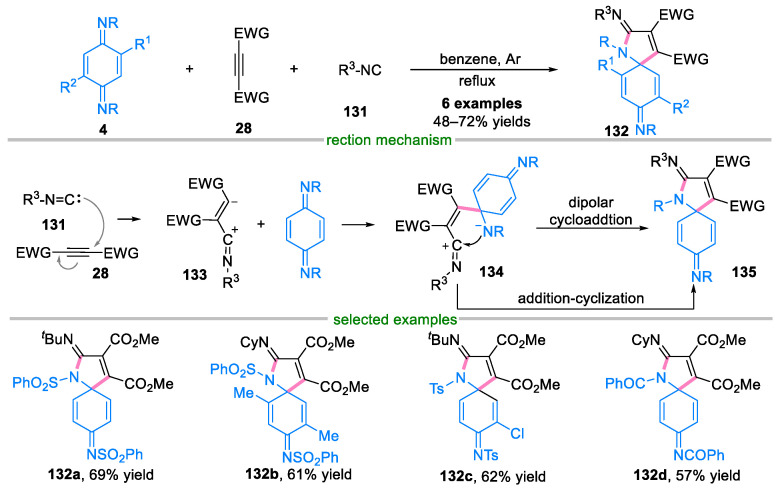
The three-component [3 + 2] cycloaddition reaction of isocyanides, DMADs, and *para*-quinone diimines.

There is only one reported case of using *para*-quinone diimines as a C–N unit to construct the spirocyclic framework. Nair et al. investigated a three-component [3 + 2] cycloaddition reaction involving *para*-quinone diamines **4**, dimethyl acetylenedicarboxylates (DMADs) **28**, and isocyanides **131** (Scheme 27) [50]. The reaction mechanism involved the nucleophilic attack of isocyanides **131** on DMADs **28**, resulting in the in situ formation of zwitterionic species **133**, and finally a 1,3-dipolar cycloaddition with the imine group of the *para*-quinone diimines to furnish γ-iminolactams **132** in up to 72% yield.

**Scheme 28:**
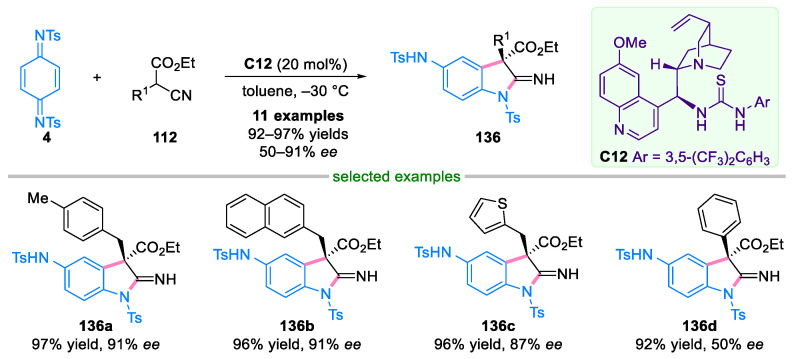
Organocatalyzed asymmetric [3 + 2] cycloaddition reaction between *para*-quinone diimides and α-cyanoacetates.

In 2018, Chandra et al. developed the first asymmetric [3 + 2] cycloaddition reaction involving *para*-quinone diimides (Scheme 28) [79]. In the presence of quinine-derived bifunctional thiourea **C12**, the α-cyanoacetates **112** first underwent nucleophilic attack to *para*-quinone diimides **4**, followed by aromatization, proton transfer, and intramolecular cyclization processes to afford chiral fused cyclic imidines **136** with up to a 91% *ee* value. Through DFT calculations, the authors suggested that multiple hydrogen bonds and tertiary amine in the chiral catalyst activated the quinone diimides and α-cyanoacetates, respectively, facilitating the interaction between the substrates and leading to the formation of the key chiral intermediates.

**Scheme 29:**
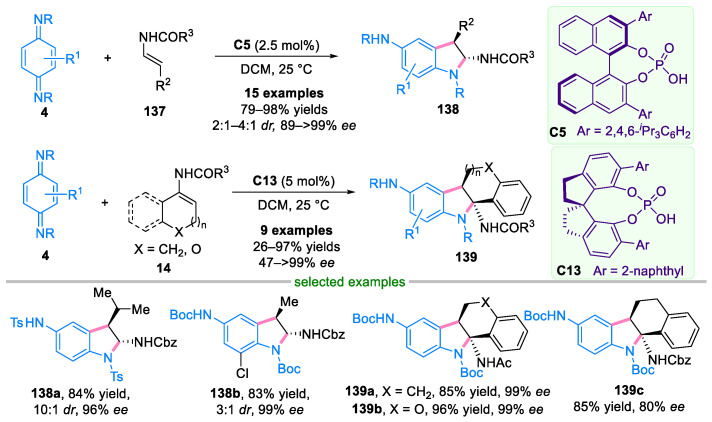
Chiral phosphoric acid catalyzed the [3 + 2] cycloaddition of *para*-quinone diimides with enamides.

Masson et al. described the synthesis of chiral 2,3-disubstituted indolines with the [3 + 2] cycloaddition of enamides and *para*-quinone diimides under the catalysis of chiral phosphoric acid (Scheme 29) [80]. For acyclic enamides **137**, chiral phosphoric acid **C5**-catalyzed [3 + 2] cycloaddition reactions provided indoline derivatives **138** in good to excellent yields with moderate diastereoselectivities and generally excellent enantioselectivities. In the presence of chiral phosphoric acid **C13**, the [3 + 2] cycloaddition reactions between cyclic enamides **14** and *para*-quinone diimides **4** showed better stereoselectivities, allowing for the formation of polycyclic compounds **139** with the highest enantiomeric excess (up to >99% *ee*).

**Scheme 30:**
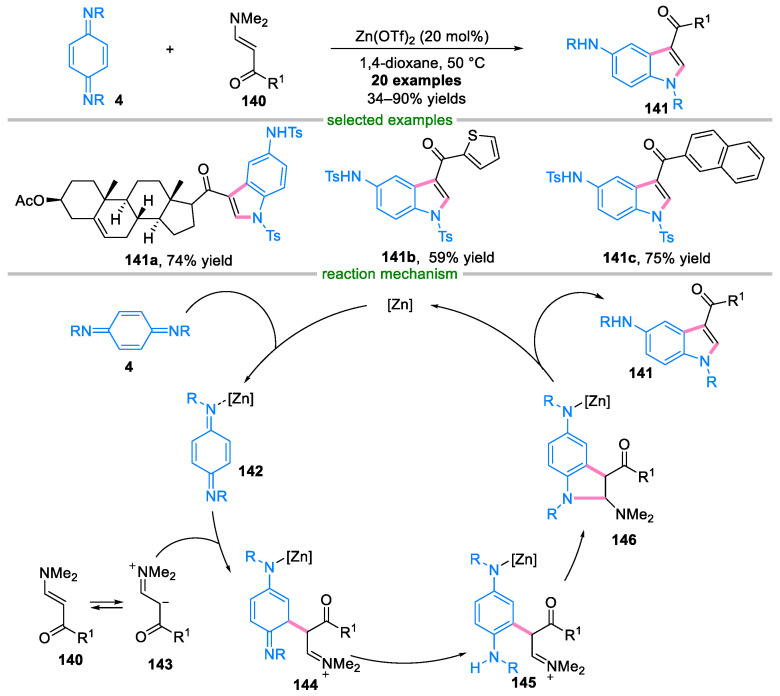
Zn(OTf)_2_ catalyzed the formal [3 + 2] cycloaddition of *para*-quinone diimides with enamides.

In 2023, Wan et al. also used enamide derivatives as the C–C unit to achieve a formal [3 + 2] cycloaddition reaction with *para*-quinone diimides (Scheme 30) [81]. Under Zn(OTf)_2_ catalysis, the isomers **143** of enamide ketones underwent a 1,4-nucleophilic addition reaction with *para*-quinone diimides **4** to give intermediates **144**. The aromatization and intramolecular cyclization reaction of intermediates **144** led to the formation of the intermediate **146**. Finally, the elimination of HNMe_2_ resulted in the formation of indoles **141** in moderate to good yields. Of note, this protocol has potential applications in the derivatization of certain natural products.

## 4. Domino Reactions of Quinone Imine Ketals

Quinone imine ketals (QIKs) have been widely used as aryl group surrogates in organic chemistry. Although Swenton et al. prepared and reported the first stable and separable QIK in 1986 [82], the low reaction selectivity caused by multiple reactive sites severely limits their application. However, with recent advances in catalytic selectivity, the use of QIKs in organic synthesis has gradually expanded. Currently, chemical transformations involving QIKs include carbon functionalization and annulation. Research on the cycloaddition reactions involving QIKs mainly includes [2 + n] annulation, formal [4 + 2] cycloaddition, [3 + 2] cycloaddition, and [5 + 3] annulation.

**Scheme 31:**
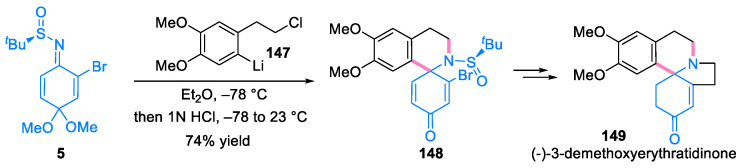
The application of chiral *N*-*tert*-butanesulfinyl QIK in the synthesis of (−)-3-demethoxyerythratidinone.

**Scheme 32:**
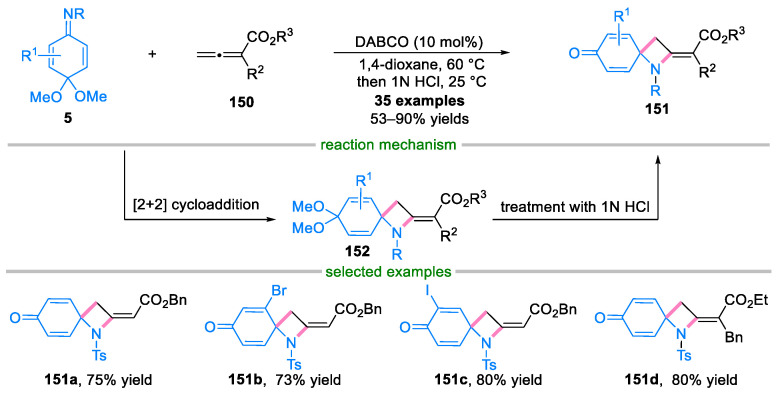
Organocatalyzed [2 + 2] cycloaddition reactions between QIKs and allenoates.

The [2 + n] annulation reactions involving QIKs include their participation as C–N units in [2 + 4] and [2 + 2] cycloaddition reactions, as 4C units in a formal [4 + 2] cycloadditions reaction, and as dienophiles in Diels–Alder reactions. Swenton and Chou first illustrated the application of QIKs as C–N units in the synthesis of natural products [83]. In 2011, Reisman et al. used the chiral *N*-*tert*-butanesulfinyl QIK **5** as a C–N unit to react with an organometallic reagent and realized the [2 + 4] annulation in 2011. With the obtained chiral product **148** as a key intermediate, they also performed the six-step enantioselective total synthesis (−)-3-demethoxyerythratidinone **149** (Scheme 31) [84]. In 2020, Cheng et al. also developed DABCO-catalyzed [2 + 2] cycloaddition reactions between QIKs **5** and allenoates **150** to generate functionalized azaspirocycles **151** in moderate to excellent yields (Scheme 32) [85].

**Scheme 33:**
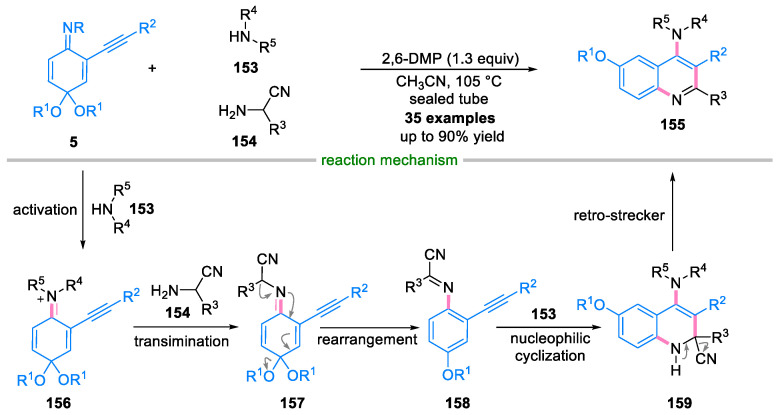
The 2-phenylethynyl QIKs-involved three-component domino reaction for the synthesis of 4-aminoquinolines. 2,6-DMP, 2,6-dimethyl phenol.

In 2016, Fan et al. used 2-alkynyl QIKs as the 4C building block to successfully developed a metal-free three-component domino reaction that resulted in a series of functionalized quinoline derivatives with yields up to 90% (Scheme 33) [86]. During the reaction process, a secondary amine **153** reacted with QIKs **5** to form intermediates **156**, which subsequently underwent transamination and aromatization to give intermediates **158**. The secondary amines **153** acted as nucleophiles on the triple bond in intermediates **158** to direct the intramolecular nucleophilic cyclization, giving intermediates **159**, followed by the retro-Strecker reaction to generate the desired products **155**.

**Scheme 34:**
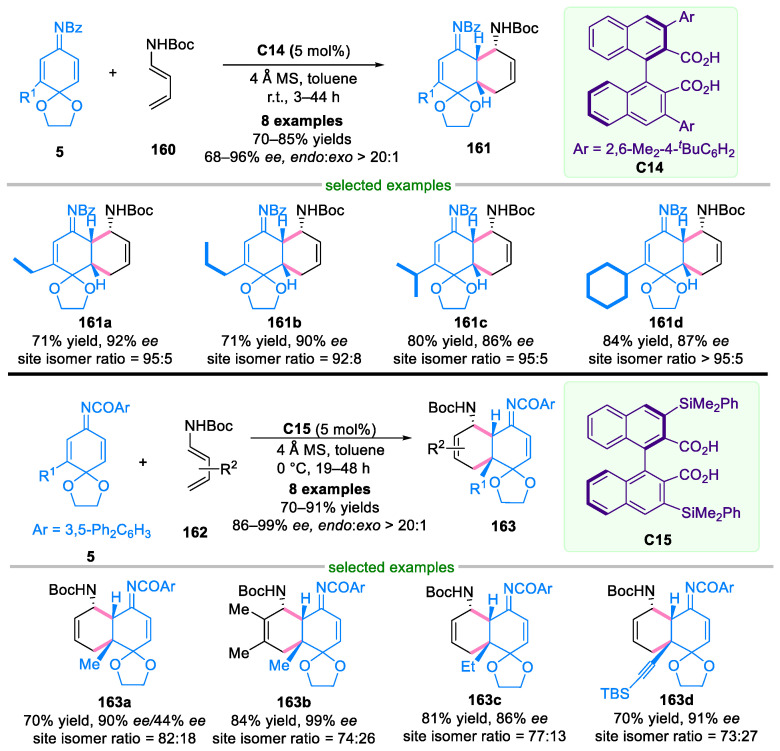
The chiral dicarboxylic acids catalyzed the Diels–Alder reaction of QIKs with dienamides.

The only reported case of Diels–Alder reaction involving QIKs was reported by Maruoka in 2015 (Scheme 34) [87]. In their study, the selection of axially chiral dicarboxylic acids as the catalyst enabled the high-yield construction of chiral cycloadducts. More importantly, when asymmetric QIKs were used in the developed transformation, changing the type of catalyst led to the selective reaction of the C=C bond. When the chiral dicarboxylic acid **C14** was used as the catalyst, the cyclization reaction took place at the unsubstituted C=C bond of QIKs, giving the corresponding products **161** in up to 85% yield and a 96% *ee* value. The chiral dicarboxylic acid **C15** promoted the reaction to occur at the more sterically hindered C=C bond, providing the cycloadducts **163** bearing a chiral all-carbon quaternary center with generally good stereoselectivities.

**Scheme 35:**
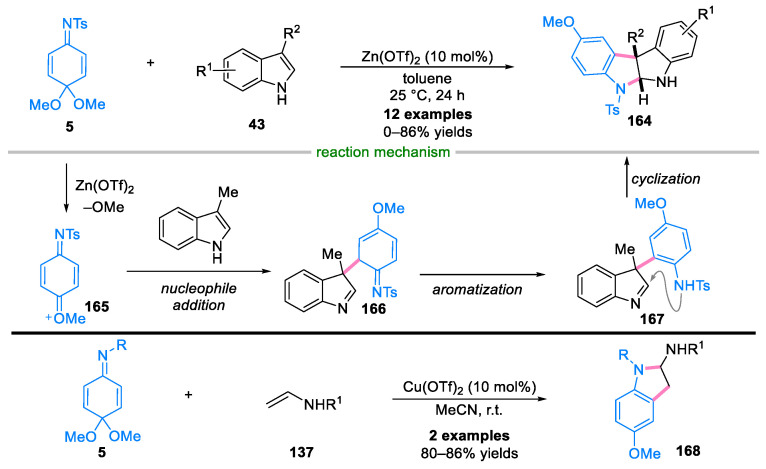
The Lewis acid-catalyzed formal [3 + 2] reaction of QIKs with 3-methylindoles and enamides.

In the study of the [3 + 2] cycloaddition reaction involving QIKs, Zhang et al. conducted extensive research and successfully constructed a series of indoline derivatives. In 2014, they first developed the formal [3 + 2] reaction between QIKs **5** and 3-methylindoles **43** (Scheme 35, top) [88]. Under the Zn(OTf)_2_ catalysis, the elimination of the methoxy group in QIK produced the quinone imine oxonium **165**, which was then subjected to nucleophilic addition by 3-methylindole to form the intermediate **166**. Subsequently, the aromatization and intramolecular cyclization led to the final product **164** in up to 86% yield. Subsequently, they also developed a Cu(OTf)_2_-catalyzed [3 + 2] annulation cyclization reaction involving acyclic QIKs **5** and enamides **137**, successfully constructing 2-carbamate-indolines compounds **168** with a maximum yield of 86% (Scheme 35, bottom) [89].

**Scheme 36:**
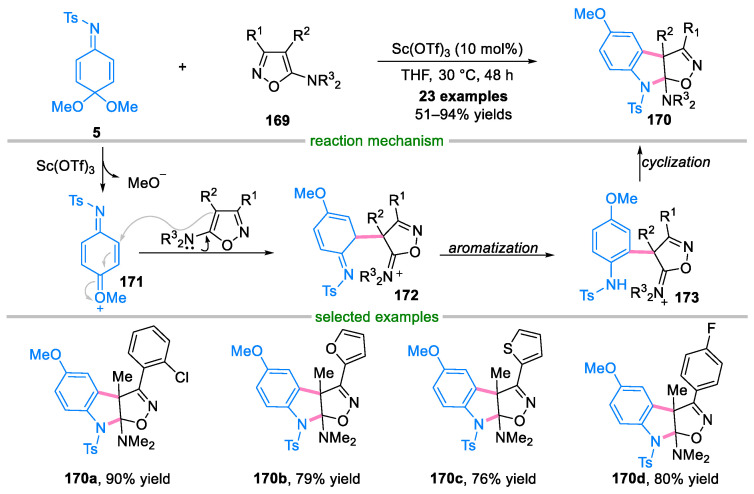
The Sc(OTf)_3_-catalyzed dearomative [3 + 2] annulation of QIK and 5-amino-isoxazolines.

In 2022, Zhang et al. also reported a Sc(OTf)_3_-catalyzed dearomative [3 + 2] annulation reaction involving QIK **5** and 5-amino-isoxazolines **169**, which led to the synthesis of a series of indoline-fused isoxazolines **170** in moderate to high yields with excellent diastereoselectivities (Scheme 36) [90]. The reported reaction mechanism is similar to their previous findings.

**Scheme 37:**
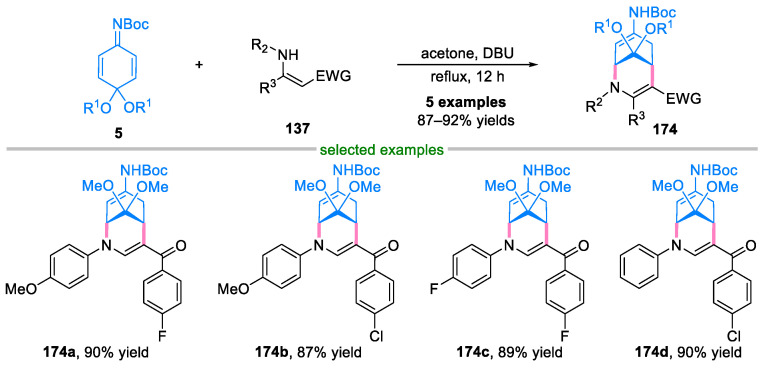
The Michael/aza-Michael addition reaction between QIKs and acyclic enamines.

**Scheme 38:**
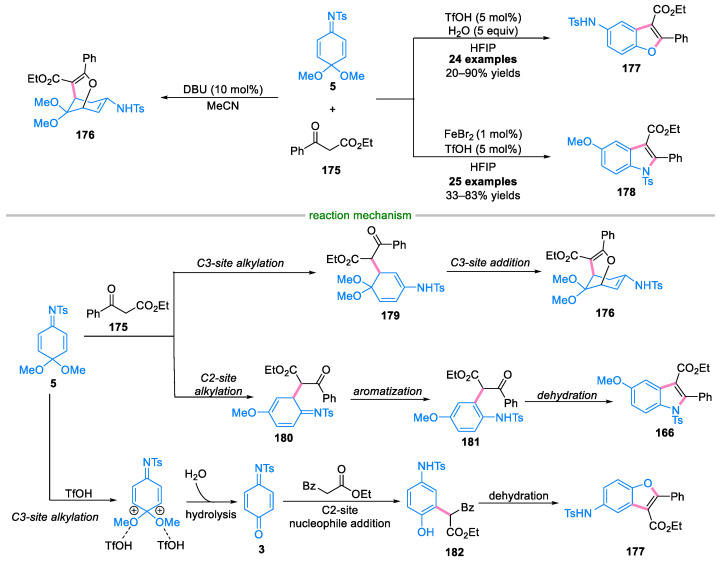
Acid-regulated divergent catalytic reaction between QIKs and 1,3-dicarbonyl compounds.

The Yan group successfully utilized QIKs **5** as 1,4-nucleophilic addition acceptors to participate in the Michael/aza-Michael addition reaction with acyclic enamines **137**, achieving the synthesis of molecularly diverse bridged ring compounds **174** in excellent yields (Scheme 37) [91]. Recently, Sun et al. performed a detailed study on the divergent transformation of QIKs (Scheme 38) [92]. By varying the type of Lewis acid and additives, they were able to achieve carbon functionalization (not shown) and annulation. Using 1,8-diazabicyclo[5.4.0]undec-7-ene (DBU) as the catalyst, cascade Michael/oxa-Michael addition reactions were successfully performed, yielding oxygen-bridged compounds **176** in excellent yields. In the presence of trifluoromethanesulfonic acid (TfOH), hydrolysis of the QIKs produced the *para*-quinone monoimines **3**, followed by the preferential attack from the β-ketoesters **175**. Subsequent aromatization led to the formation of the intermediate **182**. TfOH promoted the dehydration of intermediates **182** to give the benzofuran derivative **177**. In the absence of water, iron bromide and TfOH jointly catalyzed the reaction between β-ketoesters **175** and QIKs **5** to achieve **C2**-site alkylation and to give intermediates **180**. Subsequent aromatization and dehydration led to the formation of the indole derivatives **178**.

**Scheme 39:**
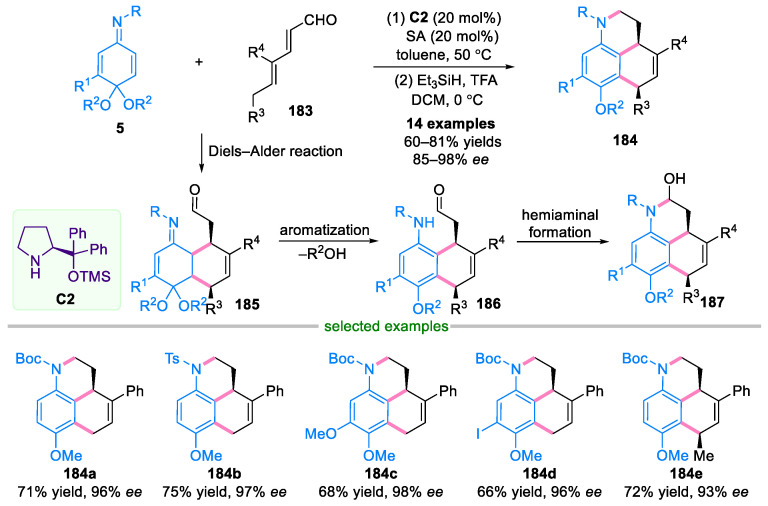
Asymmetric Diels–Alder and cascade reaction of QIKs and 2,4-dienals.

Chen et al. further explored the possibilities of using QIKs to construct chiral polycyclic compounds. In 2016, they reported a sequential asymmetric multi-step cyclization of QIKs **5** and 2,4-dienals **183** under the catalysis of proline-derived siloxane **C2**, and salicylic acid (Scheme 39) [93]. Via a domino Diels–Alder–aromatization–hemiaminal formation sequence, chiral benzo[*d*,*e*]quinolone derivatives **184** were obtained in good yields with excellent enantiocontrol. Further transformations of the products were also investigated, providing additional chiral polycyclic compounds.

## 5. Summary and Outlook

As described in this review, the widespread application of quinone imines in the efficient construction of cyclic compounds, especially nitrogen-containing heterocycles, has gained considerable attention from numerous research groups. Several quinone imines, including preformed and in situ generated quinone imines, have been designed, synthesized, and used in cyclization reactions. The use of quinone imines is widespread in the construction of polycyclic, spirocyclic, and bridged ring compounds. However, further research is required to overcome some of the remaining challenges. These challenges include, but are not limited to, the following issues. For *ortho*-quinone monoimines, their transformation only covers [4 + 2] cyclization reactions, which restricts the application of this class of compounds. Exploring other types of cyclizations to construct structurally diverse heterocyclic compounds is necessary. The cyclizations of *ortho*-quinone diimines are only used to construct five- and six-membered heterocyclic rings through [3 + 2] and [4 + 2] cyclization reactions, so it is desirable to synthesize medium-sized rings. The *para*-quinone imines are mainly selected as C–C–O(N) building blocks to participate in the [3 + n] cycloaddition reactions for the construction of oxygen- or nitrogen-containing heterocycles, but there are no reports of their involvement as C–C building blocks in annulation reactions. The QIKs exhibit versatile and flexible applications in domino reactions, including participation as a C–N unit in [2 + n] annulation reactions, serving as a C–C–N moiety for [3 + 2] cycloadditions, acting as a dual receptor for the construction of bridged ring compounds, functioning as a dienophile in Diels–Alder reactions, and enabling multi-site reactions for the construction of polycyclic compounds. However, research on asymmetric transformations involving QIKs is still quite limited. Despite the many challenges, we are confident that this area of research will reach a higher level in the coming years through the persistent efforts of chemists. We hope that this analysis will be a valuable reference for synthetic chemists interested in this field of study. The authors would also like to apologize in advance for the unintentional omission of any relevant literature report.
